# Primary Cardiac Lymphoma Presenting as a Right Ventricular Mass With Outflow Obstruction: A Diagnostic-Therapeutic Challenge

**DOI:** 10.1016/j.jaccas.2025.104929

**Published:** 2025-08-06

**Authors:** Barilee B. Abueh, Sana Rashid, Mohammad Alsheikh-Kassim, Philip Sullivan

**Affiliations:** a Internal Medicine Department, University at Buffalo, Buffalo, New York, USA; b Cardiovascular Disease Department, University at Buffalo, Buffalo, New York, USA

**Keywords:** cardiovascular disease, echocardiography, imaging

## Abstract

**Background:**

Primary cardiac lymphoma (PCL) is an exceedingly rare malignant non-Hodgkin lymphoma. It represents <2% of primary cardiac tumors often with nonspecific symptoms that may delay diagnosis.

**Case Summary:**

An 83-year-old woman with hypertension and hypothyroidism presented with progressive dyspnea and fatigue. The initial differential diagnosis was concerning for pulmonary embolism or right-heart failure. Echocardiography revealed a 6 × 7-cm right ventricular mass causing right ventricular outflow tract obstruction and right-sided heart failure. Computed tomography confirmed the presence of a mass, and biopsy identified CD20+ B-cell lymphoma. After a multidisciplinary discussion, she was started on high-dose corticosteroids with subsequent chemotherapy with R-CHOP (rituximab, cyclophosphamide, doxorubicin, vincristine, prednisone).

**Discussion:**

This case highlights the diagnostic challenge of PCL in the elderly and underscores the necessity of early multimodality imaging and coordinated care. A review of the literature shows that prompt recognition and treatment are essential for improving outcomes in this rare malignancy.

**Take-Home Message:**

Early diagnosis via comprehensive imaging and biopsy along with a multidisciplinary approach for management are all crucial steps in early detection and treatment of PCL, particularly in elderly patients.

## Graphical Abstract

**Figure:**
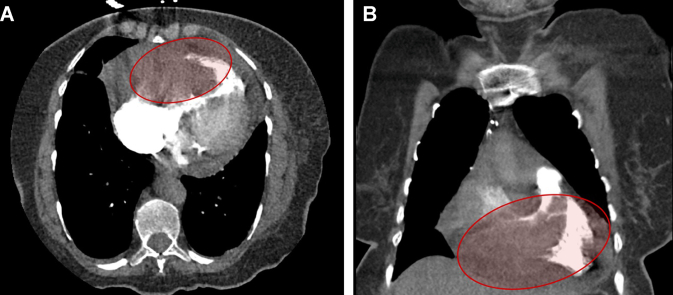

## History of Presentation

An 83-year-old woman presented with progressive dyspnea, fatigue, and exercise intolerance over 2 weeks. She reported difficulty walking short distances due to persistent fatigue. She denied chest pain, fever, night sweats, or weight loss. On examination, she appeared ill but alert, with heart rate 78 bpm, blood pressure 95/59 mm Hg, relative risk 20, and O_2_ saturation 92% on room air. Notable findings included jugular venous distension, bilateral pitting edema, a 2/6 systolic murmur along the left sternal border, and diminished breath sounds. There was no friction rub, asymmetric pulses, or embolic signs.

## Past Medical History

Her history included well-controlled hypertension and hypothyroidism. She had no known malignancy, heart failure, atrial fibrillation, thromboembolic disease, or recent infections.

## Differential Diagnosis

Primary cardiac lymphoma (PCL) was strongly suspected due to the presence of a right ventricle (RV) mass, pericardial effusion, and systemic symptoms. Metastatic cardiac tumor was part of the differential but was deemed less likely given the absence of a known primary malignancy. Intracardiac thrombus was also considered but was less probable as there was no history of atrial fibrillation, hypercoagulable state, or evidence of deep vein thrombosis or pulmonary embolism. A RV myxoma or sarcoma remained a possibility, although myxomas more commonly occur in the left atrium.

## Investigations

Laboratory findings revealed hyponatremia (128 mEq/L), mildly elevated transaminases (aspartate aminotransferase 71 U/L, alanine aminotransferase 100 U/L, alkaline phosphatase 141 U/L), and lactate of 4.2 mmol/L. B-type natriuretic peptide and high-sensitivity troponins were normal. No leukocytosis or inflammatory markers were noted.

Electrocardiogram showed a normal sinus rhythm. Chest x-ray revealed mild cardiomegaly without pulmonary edema or effusions.

**Figure 1:**
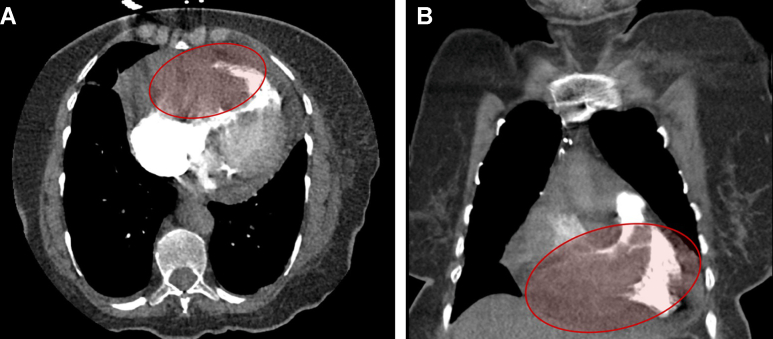
Contrast-Enhanced Chest CT Demonstrating Right Ventricular Mass (A and B) Axial view (top) and coronal view (bottom) of contrast-enhanced CT of the chest demonstrating a mass within the right ventricle (RV), extending toward the RV outflow tract. The mass appears hyperdense relative to the surrounding myocardium, suggesting a solid neoplastic process rather than a thrombus. A small pericardial effusion is noted, without significant mediastinal lymphadenopathy.

Computed tomography angiography showed a fixed RV mass encroaching on the right ventricular outflow tract (RVOT) and right coronary artery, a small pericardial effusion, and no evidence of pulmonary embolism (Figures 1A and 1B).

**Figure 2:**
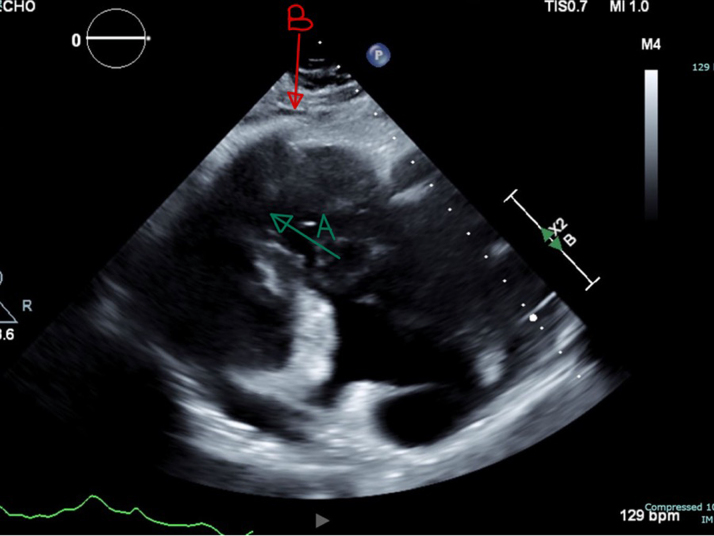
Transthoracic Echocardiography Demonstrating Right Ventricular Mass Right ventricular outflow view on transthoracic echocardiography demonstrating a large, immobile right ventricle (RV) mass (A) and occupying most of the RV cavity and extending into the right ventricular outflow tract (RVOT). The mass is echogenic and attached to the anterior RV wall (B), with no evidence of thrombus or vegetations. Findings are suggestive of primary cardiac lymphoma (PCL).

**Figure 3:**
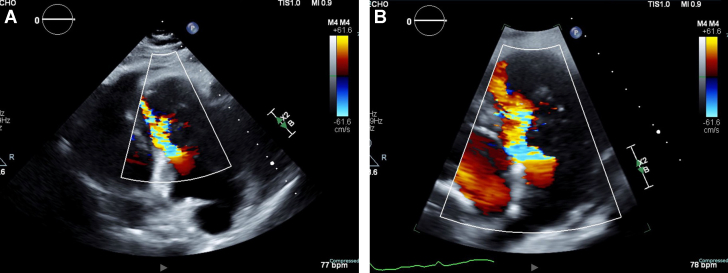
Color Doppler on Transthoracic Echocardiography Demonstrating Turbulent Flow Within the Right Ventricle and Right Ventricular Outflow Tract The aliasing pattern (multicolor flow) suggests increased velocity due to RVOT obstruction caused by the large right ventricular mass. Findings indicate hemodynamically significant obstruction due to RV mass.

**Figure 4:**
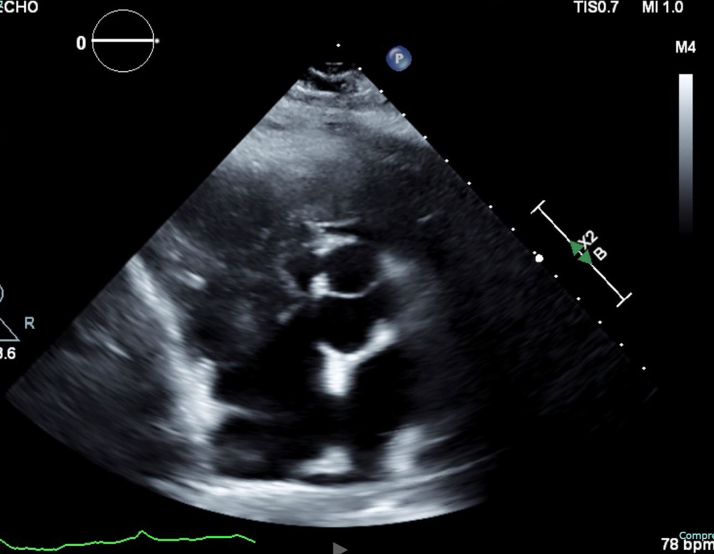
Transthoracic Echocardiogram of Subcostal Modified Short Axis Demonstrating a Large, Immobile Right Ventricular Mass Extending Into the Right Ventricular Cavity The mass appears attached to the anterior RV wall and contributes to right ventricular outflow tract (RVOT) obstruction.

Transthoracic echocardiogram confirmed a large, immobile, 6 × 7-cm echogenic mass filling most of the RV cavity and severely narrowing the RVOT. Color Doppler imaging demonstrated turbulent flow across the obstructed outflow tract, with a peak gradient measured at 9 to 16 mm Hg. Significant tricuspid regurgitation was noted. The left ventricle appeared D-shaped, suggesting increased RV pressure and volume overload. The inferior vena cava was dilated (>2.1 cm) with <50% collapsibility, indicating elevated right atrial pressure (Figure 2, Figure 3, Figure 4).

Transesophageal echocardiogram redemonstrated an immobile, echogenic RV mass attached to the anterior RV wall, with no evidence of vegetations or thrombus.

**Figure 5:**
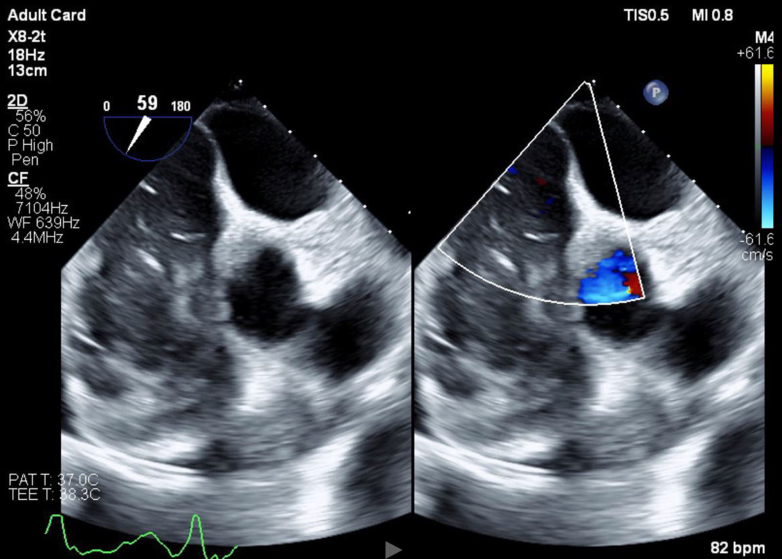
Transesophageal Echocardiogram in the Short-Axis View Demonstrating a Large, Immobile, Heterogenous, Right Atrial Mass With No Visualization of the Tricuspid Valve and Encroaching on the Superior Vena Cava The mass appears echogenic and irregular, raising suspicion for lymphomatous infiltration. The right ventricle (RV) is partially visualized.

**Figure 6:**
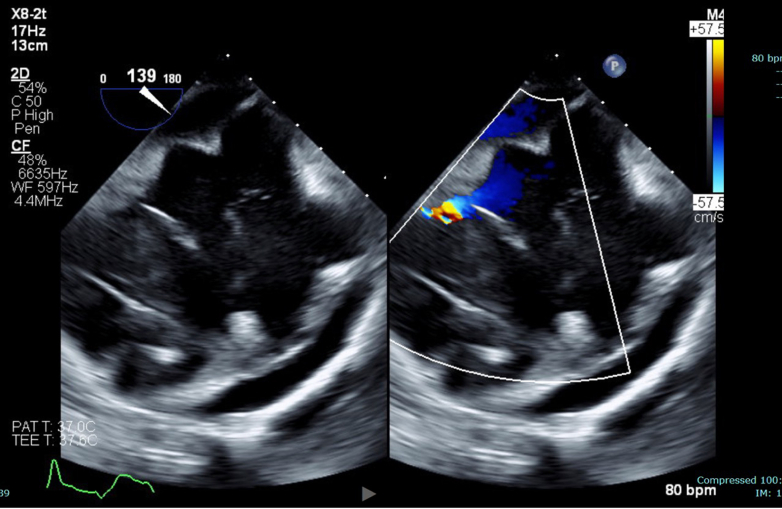
Off-Axis View of Right Atrium and Right Ventricle With Color Flow Showing Flow Acceleration in RV Shows that mass is in the RV and RA, and there is associated pericardial effusion by the RA and RV.

**Figure 7:**
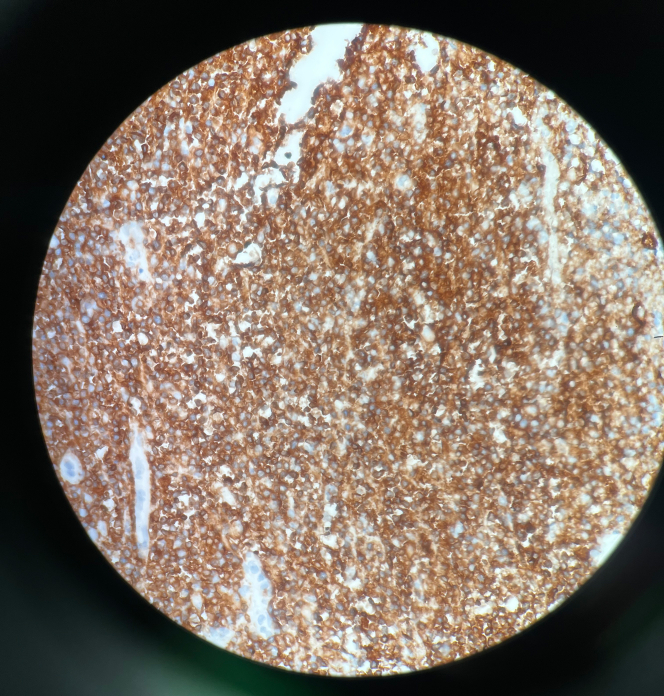
CD20 Immunohistochemistry-Positive Staining in Diffuse Large B-Cell Lymphoma This image shows diffuse membranous brown staining consistent with CD20 positivity in B cells. This supports the diagnosis of DLBCL, which typically expresses *CD20*, *CD45*, *PAX5*, *BCL2*, and *MYC*.

**Figure 8:**
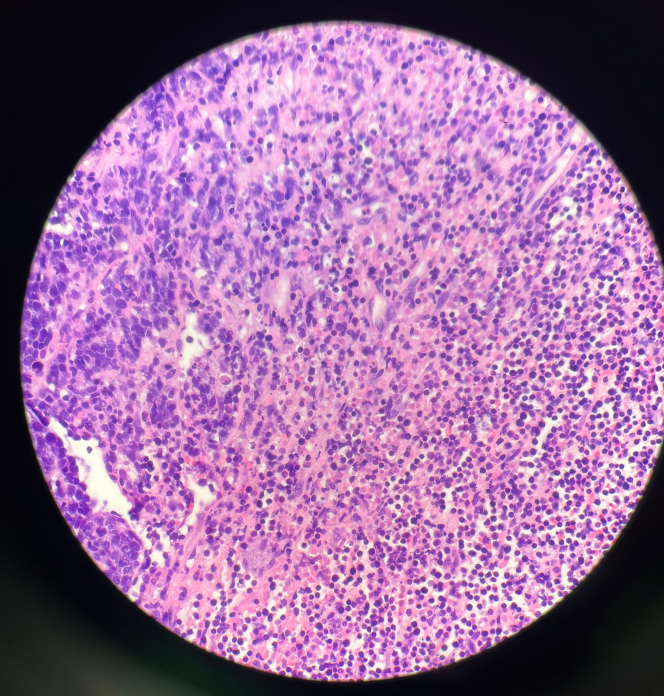
Hematoxylin & Eosin Staining Showing Diffuse Large B-Cell Lymphoma This image shows sheets of large, atypical lymphoid cells with prominent nucleoli and a high nuclear-to-cytoplasmic ratio. There is evidence of extensive necrosis present.

Endomyocardial biopsy revealed CD20+ B-cell lymphoma, confirming the diagnosis of PCL (Figures 5 and 6). Histopathology revealed extensively necrotic cardiac tissue with CD20+ large B-cell lymphoma, showing a germinal center B-cell (GCB)-like phenotype. The tumor exhibited high proliferative activity and co-expression of *BCL2* and *MYC*, consistent with a “double expressor” diffuse large B-cell lymphoma (DLBCL) (Figures 7 and 8). Fluorescence in situ hybridization analysis for *BCL2*, *BCL6*, and *MYC* genes did not identify any assay-specific rearrangements. These findings confirmed the diagnosis of PCL of the high-grade, GCB-type DLBCL subtype.

## Management

The patient was admitted to the cardiac care unit for RVOT obstruction and obstructive shock. High-dose prednisone (100 mg/d) was initiated for suspected lymphomatous infiltration, resulting in lactate improvement and symptom relief. Given the risk of hemodynamic instability, volume status was carefully managed, as excessive diuresis could lead to RV underfilling and worsening cardiac output, while fluid overload could exacerbate RV failure. Jugular venous pressure, urine output, and echocardiographic parameters guided fluid therapy.

After endomyocardial biopsy confirmed B-cell lymphoma, a multidisciplinary team involving cardiology, oncology, and critical care specialists determined that R-CHOP chemotherapy (rituximab, cyclophosphamide, doxorubicin, vincristine, prednisone) was the best therapeutic option following steroid therapy. However, after shared decision-making, the patient elected to transfer care to her local oncologist in another state. She was discharged with supplemental oxygen, transport support, and follow-up plans with oncology and cardiology.

## Discussion

Diagnosis of RV masses requires a comprehensive multimodality imaging approach. Although transthoracic echocardiography is an excellent initial tool, it is limited in its ability to provide tissue characterization and evaluation of extracardiac extension.

Transesophageal echocardiography provides higher resolution for evaluation of mass, mobility, and points of attachment, which is crucial in cases of RVOT obstruction. Anesthesia use in transesophageal echocardiogram may reduce RV preload and worsen hemodynamics, making getting images time-sensitive.^1^

Cardiac CT and cardiac MRI (CMR) enhance tissue characterization, delineate tumor margins, assess local invasion, and differentiate malignant from benign lesions.^2^ In this case, CMR was deferred as the patient was critically ill and was unable to lie flat in the scanner. Once clinically stable, she expressed a preference to pursue further evaluation and care near her home. PCL on CMR typically appears as an infiltrative soft-tissue mass that is isointense to myocardium on T1-weighted images and hyperintense on T2-weighted sequences. Post-gadolinium imaging often reveals heterogeneous late enhancement, reflecting tumor vascularity and cellularity. First-pass perfusion may show rapid enhancement, distinguishing lymphomas from thrombi.^3^ These imaging features, when combined with anatomical detail, make CMR a valuable tool in guiding diagnosis and therapeutic planning in more stable patients.

Fluorodeoxyglucose position emission tomography combined with CT can further assist in diagnosing cardiac masses based on metabolic activity and differentiating benign from malignant pathology.^4^

The endomyocardial biopsy was important in this case, revealing a DLBCL with double expression of *BCL2* and *MYC* but without chromosomal rearrangements on fluorescence in situ hybridization, consistent with an aggressive but nondouble hit subtype of DLBCL. The tumor's GCB-like phenotype and high mind bomb homolog 1 index (∼80%) indicated rapid proliferation.

In this case, suspicion of PCL was raised by the presence of pericardial effusion along with a large, immobile RV mass, which was confirmed by biopsy. Pericardial effusion is an early finding in cardiac involvement related to lymphomas and provides an important diagnostic clue when associated with the imaging features revealed by advanced techniques.^5^

The management of RV masses depends on the underlying etiology. Malignant tumors including PCL often require systemic chemotherapy or palliative care, as their invasive nature frequently precludes curative surgical resection.^6^

In this patient, high-dose corticosteroids temporarily stabilized the disease by reducing both inflammation and tumor burden, thus serving as a bridge to definitive treatment. The planned R-CHOP regimen (rituximab, cyclophosphamide, doxorubicin, vincristine, and prednisone) is supported by recent literature showing that early initiation of chemotherapy improves survival in PCL.^6^^,^^7^ One of the most important aspects of managing RVOT obstruction is the maintenance of optimal volume status. Aggressive diuresis might lead to underfilling of the RV and ultimately decrease cardiac output, while fluid overload may lead to worsening right-sided heart failure. Continuous monitoring of hemodynamics, using invasive means if available, is necessary to adapt fluid therapy appropriately.^8^^,^^9^ This was contraindicated in this situation because a Swan-Ganz catheter would go right into the mass with likely embolization.

The prognosis for PCL is poor without treatment, with survival typically lasting a few months. Early diagnosis through different imaging modalities coupled with early initiation of corticosteroids and chemotherapy can markedly improve survival. Although recent experience has improved, long-term survival is variable based on tumor biology, comorbid diseases, and the timeliness of treatment. Ongoing research into targeted therapies and advanced imaging modalities continues to offer hope for further improvements in survival and quality of life.^4^^,^^7^

## Outcome and Follow-Up

The patient showed clinical improvement with steroid therapy, particularly with decreased fatigue and dyspnea. Chemotherapy was recommended, and she chose to continue care at an oncology center closer to her home.

Although post-treatment echocardiographic or CT imaging was not available due to logistical constraints at her local facility, we maintained close communication with the patient and her healthcare proxy. She completed 6 cycles of R-CHOP. Her proxy reported initial edema attributed to cardiac dysfunction, which gradually resolved with improved mobility and tumor response. As the lymphoma regressed, her cardiac function improved, and there were no further arrhythmias or fluid accumulation. She experienced transient fevers, decreased oral intake, and brief episodes of bradycardia during chemotherapy which did not warrant pacemaker placement. Her fatigue eventually resolved, with an increase in energy and improved respiratory status. Over a 2.5-month period, she received hospital-based care for recovery and now remains under routine surveillance with her local oncology team without further hospitalizations. While the absence of follow-up imaging is a limitation, her sustained clinical improvement and reports from her proxy support a favorable treatment response.

## Conclusions

This case highlights a rare diagnosis and underscores the importance of multimodality imaging to allow for early recognition of PCL, leading to RVOT obstruction. Complex volume management needed to balance RV preload, without worsening the obstruction, as was done here, underlines the intricacies of such a case. Considering the high mortality rate associated with an untreated PCL, multidisciplinary collaboration is essential for timely intervention to optimize outcomes.

## Funding Support and Author Disclosures

The authors have reported that they have no relationships relevant to the contents of this paper to disclose.

**Take-Home Messages**

• Early diagnosis of primary cardiac lymphoma by multimodality imaging and a coordinated, multidisciplinary approach is imperative, especially in patients presenting with right ventricular masses and outflow obstruction.
• Precise volume management and expeditious initiation of high-dose corticosteroids and chemotherapy play a significant role in hemodynamic stabilization and outcome improvement.
